# How Does It Work? Unraveling the Mysteries by Which Empagliflozin Helps Diabetic and Non-diabetic Patients With Heart Failure

**DOI:** 10.7759/cureus.45290

**Published:** 2023-09-15

**Authors:** Vernicia K Hernandez, Brad T Parks Melville, Khadijah Siwaju

**Affiliations:** 1 Internal Medicine, University of Miami Miller School of Medicine, Jackson Memorial Hospital, Miami, USA; 2 Internal Medicine, University of the West Indies at Mona, Jamica, Mona, JAM; 3 Internal Medicine, University of the West Indies Cave Hill Barbados, Cave Hill, BRB

**Keywords:** cardiovascular effect, diabetic patient, sodium-glucose cotransporter-2 (sglt2) inhibitors, heart failure knowledge, empagliflozin

## Abstract

In patients with heart failure, empagliflozin offers significant cardiovascular benefits. However, its exact mode of action is unknown. Understanding the way by which empagliflozin works in heart failure may uncover additional therapeutic targets or identify other classes of drugs that may be useful to clinicians and patients. This literature review aims to unravel the mysteries by which empagliflozin reduces cardiovascular death, cardiovascular events, and heart failure hospitalization in diabetic and non-diabetic patients. Three researchers conducted the data collection. We incorporated research that used human models, animal models, patients with diabetes, and patients without diabetes. Pathology, pathophysiology, metabolism, physiology, empagliflozin, heart failure, and cardiovascular were the search terms used to probe the mesh database on PubMed. This study showed that the mechanisms by which empagliflozin could lead to positive clinical outcomes in heart failure (HF) are as follows: down-regulation of the mammalian target of rapamycin complex 1 signaling (mTORC), decreasing sarcoplasmic reticulum calcium loss, increasing cytosolic calcium loss, inducing electrolyte-free osmotic diuresis, improving fuel efficiency, and protecting the endothelial glycocalyx. These findings were inconsistent, with no generally accepted hypotheses within the scientific community. Hence we conclude that further research is required to determine the function of Empagliflozin in heart failure and the degree to which the aforementioned mechanisms of action contribute to cardiac protection.

## Introduction and background

Heart failure (HF) continues to pose a significant threat to the US healthcare system. As the population ages, the number of newly diagnosed cases, already approaching one million per year, is expected to rise [1,2]. Currently, the disease affects about 6.2 million Americans, and by 2030, this is expected to climb to about 8 million [1,2]. Since HF currently affects more than 64 million people worldwide, it is also a global threat [3]. The current five-year survival rate for HF is only 50% and 17-45% of patients die within a year of their initial admission [4,5]. Furthermore, one in 8 deaths in 2019 were due to heart failure [6]. Financial concerns are also valid. The estimated total cost of HF treatment in 2012 was 30.07 billion US dollars [7]. New evidence-based treatments are emerging as we work to increase the effectiveness and efficiency of HF treatment. Sodium-glucose co-transporter-2 inhibitors (SGLT2 inhibitors) are one such drug. The American Heart Association (AHA), American College of Cardiology (ACC), and Heart Failure Society of America (HFSA) have identified SGLT2 inhibitors as one of the four pillars in the management of heart failure as of 2022 [8]. Many clinical trials have been conducted, all of which have provided overwhelming evidence that SGLT2 inhibitors work.

The goal of the empagliflozin cardiovascular outcome event trial in type 2 diabetes mellitus patients-removing excess glucose (EMPA-REG) outcome was to assess the safety of empagliflozin (EMPA) in patients with type 2 diabetes who were at high risk of cardiovascular (CV) events. HF deaths with EMPA were calculated to be 3.7%, compared with 5.9% with placebo. Hospitalization rates were also lower in patients taking EMPA (2.4% vs. 4%) [9]. The empagliflozin outcome trial in patients with chronic heart failure and a reduced ejection fraction (EMPEROR-Reduced) trial was designed to evaluate the effect of EMPA in patients with HFrEF. Participants receiving EMPA experienced cardiovascular (CV) death and HF hospitalization at 10% and 13% respectively, compared with the placebo group which stood at 10.8% and 18.3% [10]. The empagliflozin outcome trial in patients with chronic heart failure with preserved ejection fraction (EMPEROR-Preserved) trial [11] looked at the effect of EMPA on patients with heart failure with mid-range ejection fraction (HFmrEF) and heart failure with preserved ejection fraction (HFpEF), 7.3% and 8.6% experiencing CV death and HF hospitalization respectively compared to 8.2% vs 11.8% seen in the placebo group. EMPA achieved both its primary and secondary endpoints and was shown to be superior to the placebo. Table 1 below shows landmark randomized clinical trials and their primary and secondary outcomes in patients with heart failure. 

**Table 1:** Table 1: Showing the randomized clinical trials and there primary and secondary outcomes in patients with heart failure Pts- Patients, EMPA- Empagliflozin, DAPA-Dapagliflozin, T2DM- Type 2 Diabetes Mellitus, CV- Cardiovascular, SGUT2 I- Sodium Glucose Co-Transporter 2 Inhibitors, Hosp- Hospitalization, HF-Heart Failure, HFrEF- Heart Failure with reduced ejection fraction, EF-Ejection Fraction, DECLARE-TIMI 58 - Dapagliflozin Effect on Cardiovascular Events–Thrombolysis in Myocardial Infarction 58.

Name of Clinical Trial	Year	No. of pts	Duration of Study	Name of drug	Pts without T2DM included	CV Death Placebo	CV Death SGLT2 I	Hosp % Placebo	Hosp % SGLT2 inhibitor	Conclusion
EMPA-REG OUTCOME [9]	2015	7020	37.2 months	Empa	No	5.9%	3.7%	4.1%	2.4%	SGLT2 inhibitor is superior to placebo in reducing CV events in patients with T2DM
EMPEROR- Reduced [10]	2020	3730	16 months	Empa	Yes	10.8%	10%	18.3%	13.2%	SGLT2 inhibitor is superior to placebo in improving HF outcomes in patients with HFrEF
EMPEROR- Preserved [11]	2021	5988	26.2 months	Empa	Yes	8.2%	7.3%	11.8%	8.6%	SGLT2 inhibitor is superior in reducing CV outcomes in patients with HF with EF > 40%
DECLARE- TIMI 58 [12]	2017	17160	50.4 months	Dapa	No	5.8%	4.9%	3.3%	2.5%	SGLT2 inhibitor showed reduction in HF hospitalization in T2DM patients
DAPA-HF [13]	2019	4744	18.2 months	Dapa	Yes	11.5%	9.6%	13.4%	9.7%	SGLT2 inhibitor showed reduction in CV events in patients with HFrEF

## Review

Aim of study

While there seems to be consensus on the benefit of SGLT2 inhibitors in HF patients, the mechanism of action by which it exerts this effect is not clearly understood. It is the aim of this study to examine the literature on the proposed mechanisms by which EMPA exerts its effect in improving heart failure outcomes.

Research method

To gather information for this literature review, only one database-PubMed-was used. We used the Mesh database tool, and our search terms included mixtures of the following words: pathology, pathophysiology, metabolism, physiology, empagliflozin, heart failure, and cardiovascular. We included articles that dealt with both diabetic and non-diabetic subjects, but we left out any details about other medications in the same class. No additional inclusion or exclusion criteria were used. Ninety-eight papers, all published between 2013 and 2023, were found in our search. Three researchers independently reviewed each article as part of the data-gathering process.

Discussion

Empagliflozin Suppression of the mTORC Pathway

In the study conducted by Pabel et al., myocardial tissue from patients with HFpEF exhibited decreased phosphorylation of the myofilament proteins titin, troponin I (TnI), and myosin binding protein C (MyBPC) compared to non-failing heart myocardium [14]. Patients receiving EMPA demonstrated improved titin, Tnl, and MyBPC phosphorylation. Myofilament stiffness and diastolic dysfunction in heart failure have been linked to this decreased phosphorylation of cardiac myofilament protein [14]. According to a study by Li et al., glucose transporter (GLUT) 1 and GLUT 4 receptors in the heart are where EMPA preferentially binds when compared to SGLT 1 and sodium-hydrogen exchanger (NHE) receptors. In the late stages of heart failure, increased glycolysis can negatively impact mitochondrial uncoupling, oxidative phosphorylation, and energy supply to the heart. EMPA binding to GLUT 1 and GLUT 4 may help slow the progression of the condition [5, 15]. EMPA reduction in glucose uptake down-regulates the pentose phosphate pathway via increasing Adenosine Monophosphate (AMP)-activated protein kinase leading to suppression of mammalian target of rapamycin complex 1 (mTORC) signaling [14]. The mTORC pathway is key in protein synthesis and cellular expansion and is an adaptive response to pressure overload seen in heart failure leading to cardiac hypertrophy [16, 17].

EMPA also increased the expression of the CD 36 cluster of differentiation, which aids in fatty acid oxidation and energy availability [17-19]. These beneficial effects of EMPA on pressure overload and progressive heart failure were observed in non-diabetic mice, but more study is required to fully understand the relationship between EMPA and mTORC pathway.

Empagliflozin Regulation of Calcium Within Myocytes

During heart failure, calcium (Ca2) levels increase. When Ca2 binds to troponin C it activates the actin/myosin filaments, resulting in myocardial stiffness and diastolic dysfunction in susceptible individuals. EMPA is able to combat these effects through its inhibition of the myocardial Na-H exchanger that decreases Ca and Sodium (Na) cytosol levels [20]. Another way by which EMPA modulates Ca2 is by inhibiting Calcium/calmodulin-dependent protein kinase II (CaMKII). Inhibition of CAMKII slows down the loss of calcium from the sarcoplasmic reticulum causing the myocardium in mice to contract more forcefully [21,22].

Empagliflozin Natriuretic and Diuretic Effects

Impaired diastolic function is present at an early stage of heart failure pathophysiology and is characterized by high left ventricular filling pressure and increased preload. EMPA's ability to regulate pressure inside the heart and blood vessels is one mode of action by which it may exert its beneficial cardiovascular effects in patients with heart failure. Electrolyte-free osmotic diuresis is caused by glycosuria when EMPA inhibits SGLT2 receptors on proximal convoluting tubules [23]. Hyponatremia is recognized as a poor prognostic indicator of heart failure [23-25]. In addition, compared to other diuretics, EMPA preferentially eliminates fluid from the interstitial space leading to a 5.2% increase in hematocrit seen in the EMPA study (REG OUTCOME) [24,9]. This combination of hemoconcentration and interstitial fluid contraction enhanced oxygen delivery to the myocardial tissue and decreased workload and oxygen demand [23,16].

Although the diuretic effects of EMPA are well established, the extent to which this explains the beneficial cardiac effects observed with the use of EMPA in HF treatment needs further investigation. The diuretic effects of empagliflozin make this medication dangerous in patients with a glomerular filtration rate (GFR) is less than 20mL/min/1.73 m2.

**Figure 1:**
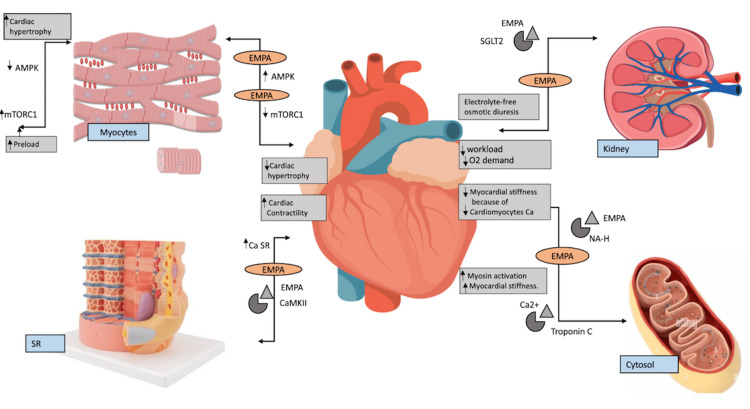
Showing the biochemical mechanisms of empagliflozin in heart failure AMP-Activated Protein Kinase, mTORC1- mTORC1 pathway, EMPA- Empagliflozin, CaSR- Calcium-Sarcoplasmic reticulum, CAMKII- Calcium/calmodulin-dependent protein kinase II, SGLT2- Sodium-glucose cotransporter-2, NA-H- Sodium hydrogenexchanger, Ca2+- Calcium

Figure 1 shows the biochemical mechanisms of Empagliflozin in heart failure.

Empagliflozin and the Efficient Fuel Hypothesis

According to the efficient fuel hypothesis, left ventricle ejection fraction (LVEF) can decrease and Left ventricle (LV) mass can increase when fuel is scarce [26]. Cardiovascular myofibrils' main energy source is (phosphocreatine system) PCr. The addition of high-energy phosphate bonds by mitochondrial creatine kinase catalyzes the conversion of Adenosine triphosphate (ATP) to PCr [27]. Mortality can be predicted by a decline in the ATP/PCr ratio [27]. A low ATP/PCr ratio is seen in patients with advanced heart failure, obesity, and hypertension [28-30]. Lack of energy is associated with impaired cardiac contractility [27]. The increase in ATP/PCr ratio seen with EMPA administration may be explained by the drug's ability to lower blood pressure and cause weight loss [31].

Santos-Gallego et al.'s findings that EMPA-treated non-diabetic pigs had increased myocardial ATP content and improved myocardial work efficiency supported this theory [32]. Comparing EMPA-treated db/db mice to placebo-treated db/db mice, [33] Abdurrachim et al. found a 45% increase in cardiac PCr/ATP. EMPA also has the capacity to increase beta-hydroxybutyrate. Beta-hydroxybutyrate enhanced cardiac performance and minimized structural remodeling in the heart in rodents. Per 100g of ketones, as opposed to 100g of glucose, ketone bodies generate more ATP [34]. However, Verma et al. assert that the alternative fuel source theory is no longer valid because in their study of the EMPA-induced 30% increase in the primary energy source of the heart ATP [35]. To what extent the efficient fuel hypothesis explains the striking cardioprotective effect of EMPA in heart failure is yet to be determined.

Empagliflozin Effects on the Endothelial Glycocalyx

Proteoglycans and glycoproteins hold the endothelial glycocalyx, a layer rich in carbohydrates that resembles a gel, to the vascular endothelium. It guards blood vessels against moving inflammatory cells, serves as a transducer for fluid shear stress, and controls nitric oxide release. Damage to the endothelium's glycocalyx from hyperglycemia results in endothelial dysfunction, which in turn causes atherosclerotic cardiovascular disease. As a result, there is increased vascular permeability in the presence of hyperglycemia as well as increased activation of the coagulation cascade and increased expression of SGLT1 and SGLT2 receptors [36]. The idea is that SGLT1 and SGLT2 receptors permit excessive glucose uptake, promoting pathophysiological structural and functional changes at the associated arterial sites [37].

Empagliflozin is thought to prevent atherosclerotic cardiovascular disease and maintain the health of the endothelial glycocalyx by bringing the level of senescence markers, including Endothelial nitric oxide synthase (eNOS), tissue factor, vascular cell adhesion molecule 1 (VCAM-1), and SGLT1 and 2, to levels comparable to those in the outer aortic arch curvature [37]. We will in fact be able to pinpoint the mechanism by which EMPA safeguards the heart as more research is done.

**Figure 2:**
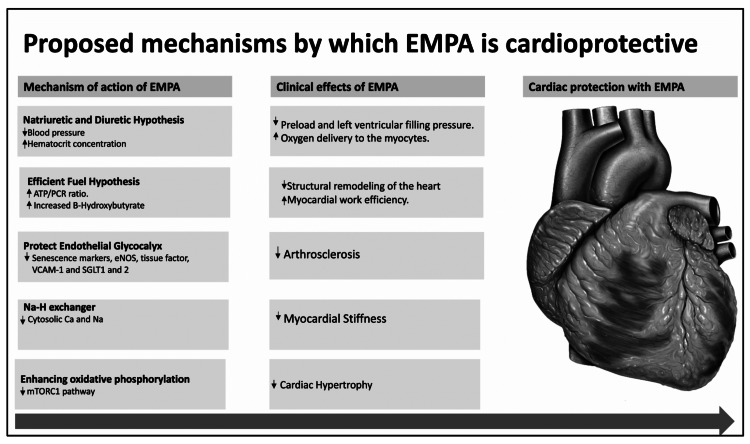
Showing the cardioprotective clinical outcomes of empagliflozin on the heart ATP/Pcr-Adenosine triphosphate and phosphocreatine system eNOS-Endothelial Nitric Oxide Synthase VCAM-1-Vascular cell adhesion protein 1, SGLT2- Sodium-glucose cotransporter-2/1, Ca- Calcium, NA- Sodium,  NA-H- Sodium hydrogen exchanger, mTORC1- mTORC1 pathway, EMPA- Empagliflozin.

Despite the long list of cardio-protective benefits of EMPA in heart failure the cost and availability of the drug pose a challenge to its use worldwide. Figure 2 shows the cardio-protective clinical outcomes of empagliflozin on the heart.

Scientific Question

Can knowledge of EMPA's mechanism of action in the treatment of heart failure aid in the identification of new therapeutic targets or identify other drug classes that may be useful to clinicians in treating heart failure?

Limitations

It is important to draw attention to this study's flaws. First off, not all potential mechanisms by which EMPA has cardioprotective effects may be covered in this research article. This is due to the investigators' bias in trying to discuss mechanisms of action that they felt were well-researched and described. Additionally, the scope of our study was restricted to a single database and a single, clearly defined search strategy. Outside of these restrictions, there may be a large number of other published and unpublished articles putting forth various possible mechanisms of action by which EMPA protects the heart in heart failure patients. Therefore, our study should be viewed as a summary of some of the numerous hypotheses that have been put forth regarding the therapeutic effects of EMPA in heart failure. It should encourage additional studies to determine how and to what extent these hypotheses account for the observed excellent clinical outcomes of EMPA in HF patients.

## Conclusions

We concluded that the mechanism by which EMPA is beneficial in heart failure remains unknown. Some proposed modes of action include suppression of the mTORC1 pathway, regulation of calcium in the sarcoplasmic reticulum and in the cytosol, electrolyte-free osmotic diuresis, improved fuel efficiency, and protection the the endothelial glycocalyx. As more studies are conducted we are confident that the mysteries by which EMPA is helpful in heart failure will be unrevealed.
